# Improving Guideline-concordant Antibiotic Use for Acute Otitis Media in a Pediatric Primary Care Network

**DOI:** 10.1097/pq9.0000000000000913

**Published:** 2026-09-04

**Authors:** Sarah Sweeney, Anna Simon, Laura Morano, Jason Chang, Kari Goebel, Kenisha Campbell, Sheryl A. Morelli, Matthew P. Kronman

**Affiliations:** From the * Department of Pediatrics, School of Medicine, University of Washington, Seattle, Wash.; † Seattle Children’s Care Network, Seattle, Wash.; ‡ North Seattle Pediatrics, Seattle, Wash.; § Department of Pediatrics, School of Medicine, University of Utah, Salt Lake City, Utah; ¶ Intermountain Health, Salt Lake City, Utah; ‖ Center for Clinical and Translational Research, Seattle Children’s Research Institute, Seattle, Wash.

## Abstract

**Introduction:**

Maintenance of Certification (MOC) projects have previously been shown to improve diagnostic accuracy and infection treatment in inpatient settings.

**Methods:**

We developed and delivered an MOC project in our ambulatory care network in 2023 to increase the use of American Academy of Pediatrics guideline-concordant antibiotic selection and duration for the treatment of acute otitis media (AOM), including amoxicillin for most patients, and the use of shorter (5–7 d) antibiotic durations in children over 2 years with nonsevere symptoms. We evaluated the use of guideline-concordant antibiotics across the network from January 2021 to September 2025, including periods before, during, and after the MOC intervention. We compared the use of guideline-concordant antibiotics between clinics that participated in the MOC intervention and those that did not.

**Results:**

The study period included 40,329 AOM episodes. By the end of the study period, 64.7% of children with AOM across the network received guideline-concordant antibiotic selection and duration, up from 46.6% at baseline. After the intervention, MOC-participating clinics had increased use of guideline-concordant antibiotic selection and duration compared to nonparticipating clinics (70.1% versus 54.5%). The frequency of patients returning within 7 days for a repeat AOM visit did not differ between the baseline period (124/12,735; 1.0%) and the postintervention period (303/27,594; 1.1%) (*P* = 0.3).

**Conclusions:**

An MOC program was effective in increasing guideline-concordant antibiotic prescriptions for pediatric AOM across an outpatient network. This effect persisted over time and was stronger in MOC-participating clinics. MOC participation has the potential to improve antibiotic stewardship in ambulatory settings.

## INTRODUCTION

### Problem Description

Acute otitis media (AOM) is the most common ambulatory childhood infection for which children receive antibiotics.^1,2^ Approximately 4% of children with AOM treated with antibiotics have follow-up visits for adverse effects, but 32%–45% report adverse effects when interviewed using validated quality-of-life measures. Children who received broad-spectrum antibiotics are more likely to experience adverse events than those treated with narrow-spectrum antibiotics.^3^

The American Academy of Pediatrics (AAP) Red Book includes a systems-based treatment table outlining age-based recommendations for antibiotic prescribing for AOM, including 7 days of amoxicillin for children between 2 and 5 years of age and 5 days for children 6 years and older, durations shorter than those used historically. The AAP continues to recommend 10 days of amoxicillin for children younger than 2 years and those with severe symptoms.^4^ Previous studies demonstrated that a minority of pediatric outpatient antibiotic prescriptions are optimal for both antibiotic selection and duration. A 2024 study in Tennessee found that across almost 500,000 encounters involving children of all ages, only 38.5% of prescriptions were optimal for antibiotic selection, 51.3% for antibiotic duration, and 31.4% for both antibiotic selection and duration.^5^ Preliminary data from our training hospital-affiliated primary care clinic network showed that approximately 49% of patients with AOM received optimal antibiotic selection and duration in 2021.

To ensure pediatricians remain current with the latest guidelines, they must participate in the Maintenance of Certification (MOC) program, a formal process established by the American Board of Pediatrics (ABP) to update their clinical knowledge and skills over time. To maintain board certification, pediatricians certified after 1988 must complete the MOC program every 5 years.^6^ MOC consists of four components: “Professional Standing and Licensure (part 1),” referring to the maintenance of a valid, unrestricted medical license and compliance with all ABP policies, rules, and codes; “Lifelong Learning (part 2),” referring to the assessment and enhancement clinical knowledge and skills; “Exam (part 3),” an assessment to measure knowledge; and “Health Care Improvement (part 4),” referring to the assessment and improvement of patient care quality and processes that will lead to improved child health and healthcare. The objectives of parts 2 and 4 are primarily achieved by earning points through a wide variety of ABP-approved activities and projects.^7,8^

### Available Knowledge

Previous studies evaluated the effectiveness of quality improvement (QI) interventions like plan-do-study-act (PDSA) for optimizing infection detection and reducing broad-spectrum antibiotic use in ambulatory settings,^2,9,10^ improving the use of safety net antibiotic prescriptions in ambulatory settings,^11^ optimizing antibiotic duration in emergency departments^12^ and urgent care centers,^13,14^ and reducing overall antibiotic use in the neonatal intensive care unit.^15^ To our knowledge, however, no studies have demonstrated the effectiveness of MOC projects in optimizing both antibiotic selection *and* duration for AOM in the primary care setting.

### Specific Aims

We developed a QI project within our primary care clinic network to improve antibiotic selection and duration for children diagnosed with AOM. The project offered ABP MOC Part 4 credit as an incentive to participating physicians and was available to all network practices. At the MOC project outset, our project goals included (1) reducing antibiotic prescribing duration for pediatric patients diagnosed with AOM by 10% for all ages and by 25% for children over age 2 years and (2) increasing the use of amoxicillin versus other antibiotics by 10% overall for pediatric patients with AOM. Here, we described our project and the associated changes in antibiotic prescribing.

## METHODS

### Context

The Seattle Children’s Care Network (SCCN) is a clinically integrated pediatric network in our geographic region, a collaboration between clinicians and administrators to improve quality, reduce costs, and enhance patient experience across the care continuum. During the study period, the SCCN comprised 16 individually owned primary care practices with 21 locations across the Puget Sound region of Washington state.^16^ Practices have independent electronic medical records (EMRs) but contribute patient data (demographics, allergies, and diagnoses) and prescription data to the SCCN. Only 2 of the practices were directly affiliated with academia during the study period, and neither of these practices enrolled in the MOC project. The MOC-enrolled and non-MOC-enrolled practice groups were similar in geographic distribution and in the mix of rural and urban practices. We collected individual practice data in a population health tool (Arcadia, Boston, Mass.). Each primary care health record is connected to the population health tool, importing clinical and billing information daily. We also included additional claims data from insurers’ value-based contracts in the population health tool dataset. Data are validated and aggregated in the tool when each data connection is implemented.

### Planning the Intervention

The SCCN Quality and Care Transformation Committee aims to include a participating member from each SCCN practice, as well as representatives from the SCCN Board of Directors and administration. The SCCN Quality and Care Transformation Committee reviewed proposed MOC projects to launch within the SCCN and selected the AOM antibiotic stewardship project as one with high clinical practice relevance, importance for patients, and opportunity to improve the quality of care while reducing costs.

### Improvement Team

The core members of the improvement team included the SCCN Program Manager (K.G.), Data Analysts (J.C. and A.S.), Chief Medical Officers (S.M. and K.C.), Care Transformation consultant (L.M.), and a Pediatric Infectious Diseases physician with expertise in outpatient antimicrobial stewardship (M.K.). Participating practices identified local physicians, nurses, and other team members to participate in the improvement work.

### Interventions

The SCCN improvement team applied to participate as an MOC project through the Seattle Children’s Hospital MOC program in 08/2022; the MOC program approved the project in September 2022. We announced the project to the SCCN Quality and Care Transformation Committee in September 2022, in the SCCN email newsletter to all practices in November 2022 and December 2022, and during a 30-minute educational webinar on antibiotic stewardship in December 2022, offered in conjunction with the Centers for Disease Control and Prevention’s annual Antibiotic Awareness Week.^17^ Physicians at each practice could independently elect to join the MOC project.

**Fig. 1.**
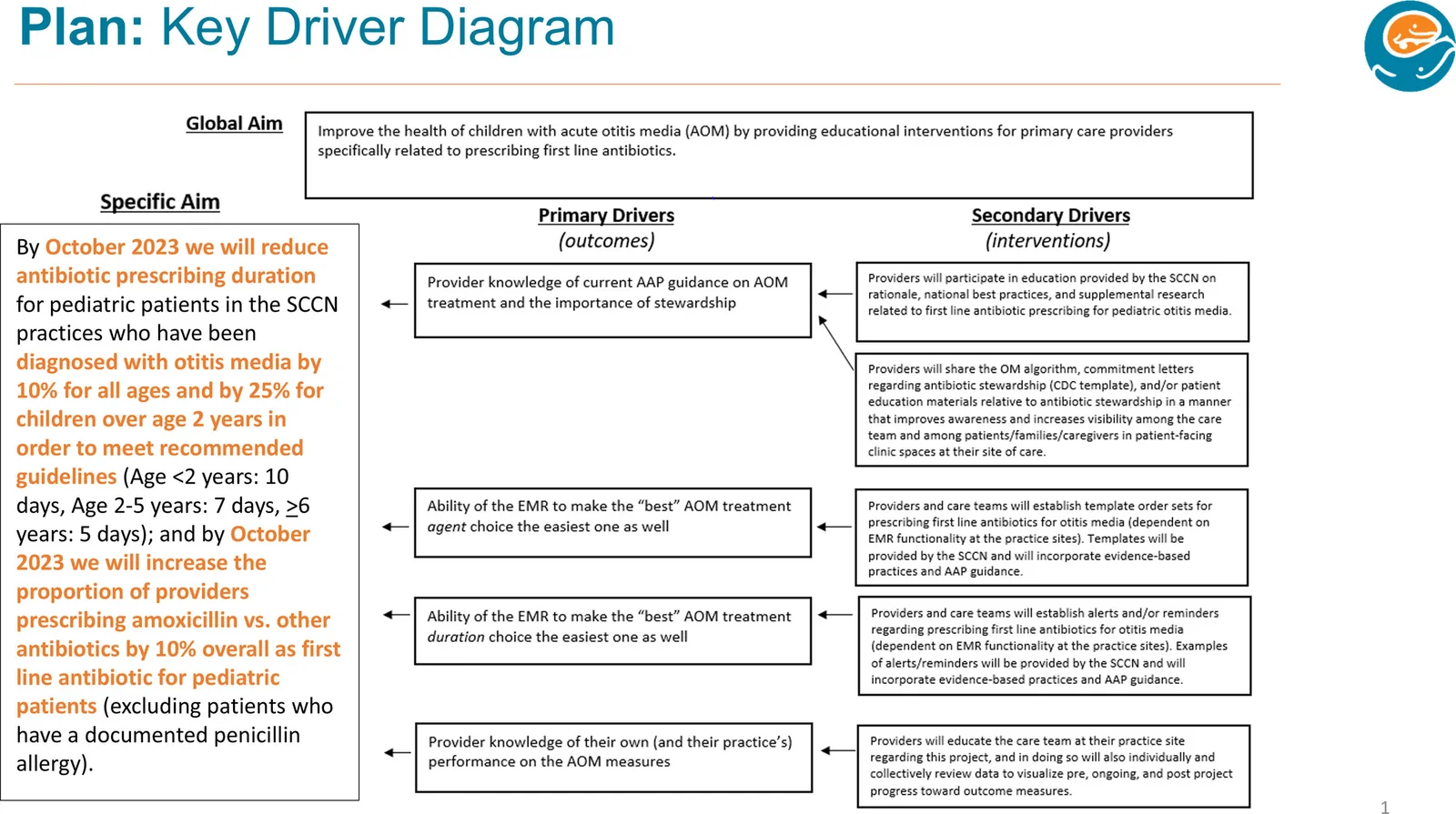
Key driver diagram for improving the quality of antibiotic prescribing for AOM across the network.

We offered MOC kick-off meetings to physicians from participating practices on May 1, 2023, and December 1, 2023, at different times to accommodate the schedules of identified clinicians and key drivers of improved prescribing for AOM (Fig. 1). During the kick-off meetings, we provided all participants with updated education on antibiotic prescribing recommendations per the AAP guidelines and the durations of therapy recommended in the AAP Red Book,^4^ including that amoxicillin is the first-line choice for most patients, and that the age-based first-line durations of therapy for children without severe symptoms are 10 days for children under 2 years, 7 days for children 2–5 years, and 5 days for children 6 years and older. We also offered all participants a webinar training in February 2023 on evidence-based best practices for communicating about antibiotic prescribing.^18^

The intervention team created, posted, and updated the Antibiotic Stewardship toolkit available on the SCCN internal website. The website also serves as a forum for peer support, enhancing learning and enabling the sharing of best practices across sites. The MOC improvement team met with each practice individually for kick-off, interim, and project closure meetings to discuss each practice’s specific interventions, with the final project closure meetings occurring in November 2023. During these meetings, the improvement team provided implementation coaching for team-based engagement and consistent messaging and reviewed practice-specific prescribing data. Interventions could vary across practices and were driven by each practice’s internal review of local drivers and barriers to optimal care, which typically identified a lack of knowledge of the full AAP guidelines and EMR-based systems as causes of suboptimal prescribing. Common interventions across the practices included updating practice EMR prescribing preference lists, encounter templates, documentation macros, and pop-up alerts, or providing additional education to one another within the clinic on AOM prescribing.

### Study of the Intervention

We obtained data from January 1, 2021 to September 30, 2025, covering the periods before, during, and after the MOC intervention. We measured the effects of the intervention on children aged 6 months–18 years who were diagnosed with AOM, prescribed an antibiotic, and were current patients in a primary care practice affiliated with the SCCN. We confirmed AOM diagnoses using billing codes (**see eTable 1, Supplemental Digital Content,** https://links.lww.com/PQ9/A798). We excluded patients with immunodeficiency, those who had received antibiotics within 30 days of the AOM visit, or those with a secondary diagnosis code for an additional infection that could alter antibiotic prescribing (such as cellulitis or streptococcal pharyngitis). Because diagnosing AOM typically requires a physical examination of the ear, we excluded patients seen via telemedicine to minimize misclassification of AOM visits. We excluded patients in whom the antibiotic duration exceeded 14 days, as this could indicate data quality issues or ongoing treatment of other infections not captured in the diagnosis codes, because AOM is seldom treated for longer than 14 days. We collected prescribing events in which the agent used was amoxicillin, amoxicillin/clavulanate, cefdinir, azithromycin, or trimethoprim-sulfamethoxazole. For antibiotic selection, we excluded patients with an allergy to amoxicillin. For antibiotic duration measures, we excluded patients prescribed azithromycin because it is almost exclusively administered as a 5-day course.

### Measures

Our primary outcome was the proportion of AOM patients prescribed an antibiotic in alignment with the AAP-recommended guidelines for antibiotic selection (amoxicillin) and duration by age, as noted above. Because each participating clinic functions independently and uses its own EMR, a network-wide process measure for the intervention is unavailable. As a balancing measure, we examined all-cause return visits and AOM-specific return visits within 7 days of treatment initiation, including both in-person and telemedicine visits.

### Analysis

During the MOC project, we shared dashboards showing data trends with each participating clinic. Although providers could enroll in the MOC project independently, multiple providers commonly enrolled within the same practice and developed clinic-level QI interventions. For this reason, we analyzed the data by practice, comparing those with at least one provider enrolled in MOC to those with no providers enrolled in MOC. We provided control P-charts demonstrating the proportion of AOM patients who received the optimal antibiotic selection and duration across the entire cohort and stratified by SCCN practices participating in the MOC project versus those not participating. We presented these data monthly, with three-sigma limits used to set the upper and lower control limits, and standard rules for identifying special-cause variation, including 8 or more values above the baseline. On the P-charts, we noted the beginning of the MOC intervention (January 2023), the end of the MOC intervention (October 2023), and Centers for Disease Control and Prevention Antibiotic Awareness Week (November 2024), during which all practices reviewed data on improvements in antibiotic prescribing associated with the 2023 MOC project. We used the chi-squared test to compare the performance of MOC and non-MOC participants; we considered *P* < 0.05 significant. We assembled control charts per the Institute for Healthcare Improvement guidelines.^19^ We reported this QI initiative in accordance with the Standards for QI Reporting Excellence Guidelines.^20^

### Ethical considerations

The Seattle Children’s institutional review board did not consider this QI work to be human subjects research, and it was thus exempt from review.

## RESULTS

**Fig. 2.**
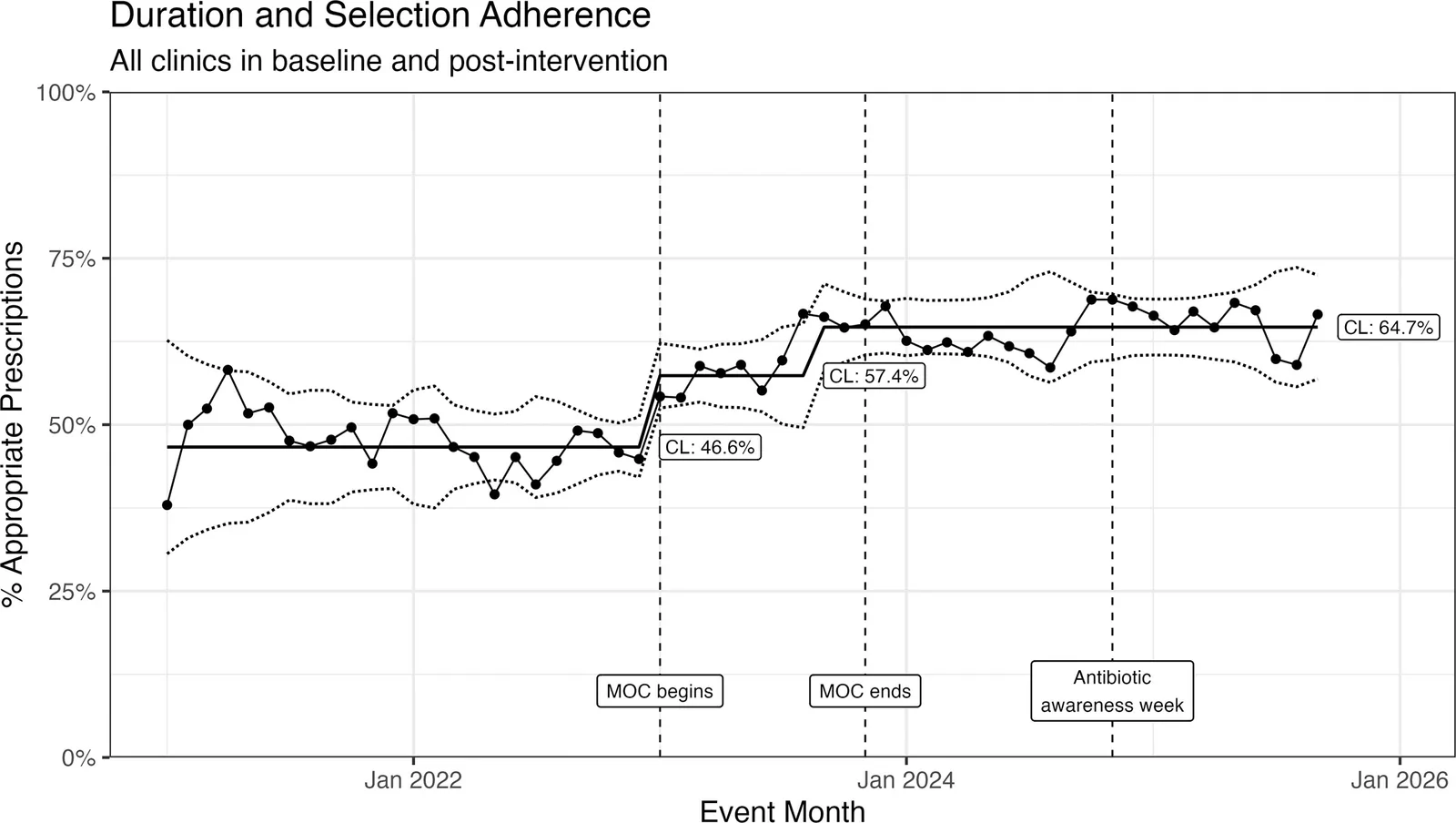
P-chart showing the proportion of patients receiving optimal antibiotic selection and duration for AOM over time.

**Fig. 3.**
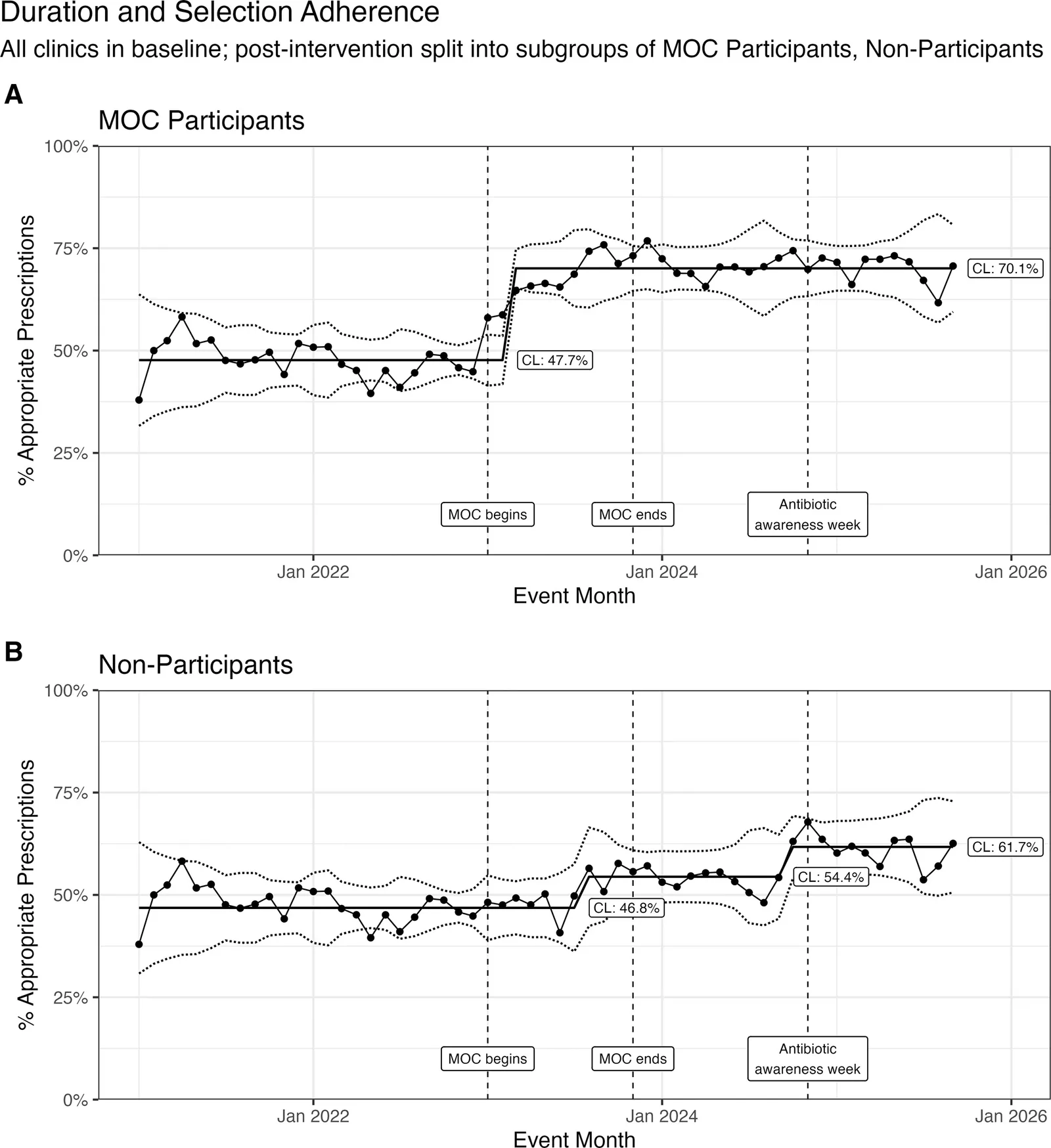
P-chart showing the proportion of patients receiving optimal antibiotic selection and duration for AOM over time, divided into the subgroups of having an AOM visit at an MOC-participating clinic (A) or a non-MOC-participating clinic (B).

**Fig. 4.**
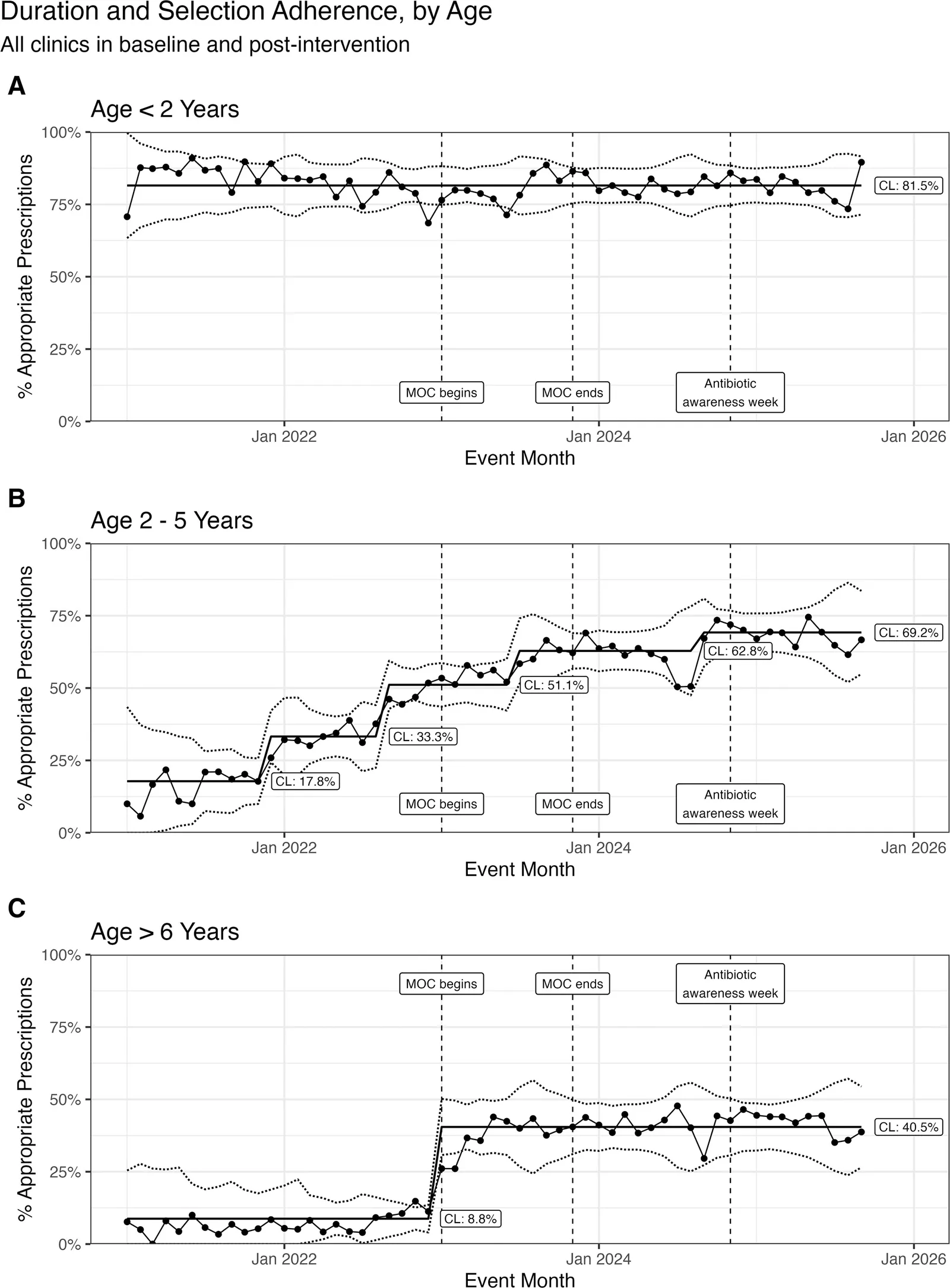
P-chart showing the proportion of patients receiving optimal antibiotic selection and duration for AOM over time, divided into the subgroups of age <2 years (A), age 2–5 years (B), and age >6 years (C).

During the study period, 40,329 AOM visits occurred at the 16 included clinics (Table 1). During the intervention period, the mean proportion of children of all ages who received antibiotic selection and duration for AOM increased, from a mean of 46.6% before the MOC project to 64.7% after the conclusion of the MOC project, which was sustained for almost 2 years after the conclusion of the project (Fig. 2). When stratifying the cohort by whether children were seen in MOC-enrolled versus non-MOC-enrolled clinics, the improvement in antibiotic selection and duration was more pronounced in MOC-enrolled clinics at the end of the MOC program (70.1% versus 54.4%, Fig. 3). The improvement over time in the use of optimal antibiotic selection and duration was greater in children 2–5 years and >6 years of age than in children <2 years (Fig. 4), because baseline use of optimal antibiotic selection and duration was high among children <2 years.

**Table 1.** AOM Encounters of Included Subjects by Age and Clinic Group

	Baseline	PostIntervention		Total
		Non-MOC-enrolled Clinics	MOC-enrolled Clinics	
Encounters for children <2 y old	4,599 (36.1%)	4,422 (34.5%)	4,791 (32.4%)	13,812 (34.2%)
Encounters for children 2–5 y old	5,309 (41.7%)	5,286 (41.3%)	5,539 (37.5%)	16,134 (40.0%)
Encounters for children >6 y old	2,827 (22.2%)	3,098 (24.2%)	4,458 (30.1%)	10,383 (25.8%)
Total encounters for children of all ages	12,735	12,806	14,788	40,329

During the baseline period, 124 of 12,735 (1.0%) patients returned within 7 days of antibiotic initiation for AOM. After the start of the intervention, 303 of 27,594 (1.1%) of patients returned within 7 days (*P* = 0.3 comparing baseline and postintervention). The occurrence of these visits differed between non-MOC-enrolled and MOC-enrolled clinics in the postintervention period (0.9% versus 1.3%, *P* = 0.001).

## DISCUSSION

In this retrospective cohort of over 40,000 AOM patient visits over 57 months, we found that the MOC program increased the use of guideline-concordant antibiotic selection and duration across the network, and that MOC-participating clinics experienced greater improvement than non-MOC-participating clinics. Furthermore, these improvements persisted throughout the study, with guideline-concordant practice rates remaining elevated at 32 months after MOC initiation. Guideline-concordant antibiotic use among children under age 2 did not improve over the study period, as these rates were already high at the study’s outset. Importantly, the frequency of return visits for AOM, a balancing measure designed to capture potential unintended consequences such as complications or treatment failure, did not increase during the study’s postintervention period. There was a statistically significant increase in return visits from the MOC-participating clinics (1.3%) to the non-MOC-participating clinics (0.9%) in the postintervention period. Still, this increase was small (0.4%) and driven by the study size, so it may not be clinically meaningful. This MOC project accomplished our goals of reducing antibiotic prescribing duration for pediatric patients diagnosed with AOM by >10% across all ages (Fig. 2) and by >25% for children over age 2 years (Fig. 4). Still, it did not achieve the goal of increasing the use of amoxicillin relative to other antibiotics by 10% overall, due to high baseline rates of amoxicillin use in the network (agent-specific data not shown).

For children 2–5 and those >6 years of age, we observed increases in guideline-concordant antibiotic selection and duration across the network. Beyond improvements in antibiotic use due to MOC participation, other possible explanations for the increases observed in the non-MOC-participating clinics include broader initiatives beyond the MOC (eg, other didactic interventions, updated guidelines, national or regional campaigns, media coverage, policy changes, or institutional priorities). After the MOC project began, prescribing in both groups coincided with known national amoxicillin shortages. This fact should not have affected the differences between the 2 study groups.

Our findings align with prior literature demonstrating the efficacy of QI interventions in increasing guideline-concordant clinical care. Although previous studies have investigated the effectiveness of MOC projects in optimizing infection detection and antibiotic selection, this is the first study to identify a significant increase in guideline-concordant antibiotic prescription in terms of selection and duration for the treatment of AOM in the primary care setting after MOC participation. In 2018, investigators evaluated QI interventions paired with ABP MOC Part 4 credit to reduce unnecessary rapid streptococcal testing and inappropriate antibiotic use in an ambulatory, training hospital-affiliated clinic.^9^ The intervention successfully reduced unnecessary testing from 64% to 40.5% but did not yield a statistically significant improvement in appropriate antibiotic use for streptococcal pharyngitis; however, inappropriate antibiotic use was primarily related to unnecessary testing. In 2022, investigators implemented an MOC project in the emergency departments and urgent cares of a large urban training hospital, pairing QI methods with ABP MOC Part 4 credit to improve antibiotic selection and shorten the duration of antibiotic therapy for children seen with purulent and nonpurulent skin and soft tissue infections.^13^ In 2013, Gerber et al described the efficacy of an audit-and-feedback system in reducing the use of broad-spectrum antibiotics in outpatient pediatric primary care clinics.^21^ The intervention was effective; however, after the audit-and-feedback system was removed, the benefits were lost.^22^ This outcome contrasts with our findings that MOC participation led to sustained benefits over 32 months after MOC initiation. The sustained benefits of our MOC may be attributable to the “Ikea effect,” a behavioral economics concept that refers to people placing higher value on a product to which they contributed time and effort.^23^ In addition, many of the interventions in our study involved permanent changes (eg, to the EMR),^15^ the educational and audit-and-feedback intervention used in other QI studies.

Previous studies have also described the effectiveness of other QI interventions, such as PDSA, in increasing appropriate antibiotic use for AOM across a variety of settings. In both the urgent care and emergency department settings, the use of PDSA cycles, including education and EMR changes, improved adherence to AOM treatment duration guidelines.^12,14^ However, our study differs in that these studies only evaluated children older than 2 years, had limited demonstration of sustained effect, and did not evaluate the role of MOC as an incentive for driving improvement. A 2019 study compared rates of guideline-concordant antibiotic selection for AOM in cohorts that did and did not undergo intervention and found that three PDSA cycles improved rates of narrow-spectrum antibiotic selection in pediatric primary care clinics; however, this study did not examine antibiotic duration. Rather than through voluntary participation, the cohorts were identified based on clinics’ baseline adherence rates to antibiotic selection guidelines; only clinics with <90% adherence received the intervention.^10^

Our study has limitations. Because interventions could differ across the included practices, tracking individual practice-based process measures was not feasible. Increases in guideline-concordant prescriptions may have been influenced by secular trends in antimicrobial stewardship outside the MOC project. Providers inclined to participate in the MOC project may have been more motivated to practice evidence-based medicine, which could have affected the association between MOC participation and improvements in antibiotic prescribing. Our 7-day time frame for return visits, as a balancing measure, may have been too brief to adequately capture any harm from a shorter antibiotic duration. Our study only captures the management of index episodes of AOM and does not track the management of AOM treatment-failure episodes. Finally, some clinics experienced provider turnover during the study window, which could introduce sampling error.

## CONCLUSIONS

MOC participation was effective in significantly increasing guideline-concordant antibiotic selection and duration for pediatric AOM in the outpatient setting. Furthermore, this effect persisted over time. This study, in concert with previous studies,^3,9,13,15^ underscored the potential for MOC participation to increase guideline-concordant practice across a variety of conditions and settings, thereby demonstrating its importance in driving high-quality care.

## ACKNOWLEDGMENTS

The authors would like to acknowledge the following primary care practices that enrolled in this MOC project for their exemplary efforts: Bainbridge Pediatrics, Northwest Pediatric Center, Olympia Pediatrics PLLC, Pediatric Associates of Whidbey Island, Skagit Pediatrics, South Sound Pediatrics and Woodinville Pediatric Clinic. The authors also acknowledge the extremely helpful assistance of the MOC program at Seattle Children’s Hospital.

## Supplementary Material


