# Supplementary material for: Improving Guideline-concordant Antibiotic Use for Acute Otitis Media in a Pediatric Primary Care Network
Source: Pediatr Qual Saf. 2026 Sep 4;11(5):e913. doi: 10.1097/pq9.0000000000000913 (PMC13544754; doi:10.1097/pq9.0000000000000913)
Supplement: Supplementary file 1 [file pqs-11-e913-s001.pdf]

eTable 1: ICD-10 codes used to identify acute otitis media cases

H65.00	Acute serous otitis media, unspecified ear
H65.01	Acute serous otitis media, right ear
H65.02	Acute serous otitis media, left ear
H65.03	Acute serous otitis media, bilateral
H65.04	Acute serous otitis media, recurrent, right ear
H65.05	Acute serous otitis media, recurrent, left ear
H65.06	Acute serous otitis media, recurrent, bilateral
H65.07	Acute serous otitis media, recurrent, unspecified ear
H65.111	Acute mucoid otitis media of right ear
H65.112	Acute mucoid otitis media of left ear
H65.113	Acute mucoid otitis media of both ears
H65.191	Other acute nonsuppurative otitis media, right ear
H65.192	Other acute nonsuppurative otitis media, left ear
H65.193	Other acute nonsuppurative otitis media, bilateral
H65.194	Other acute nonsuppurative otitis media, recurrent, right ear
H65.195	Other acute nonsuppurative otitis media, recurrent, left ear
H65.196	Other acute nonsuppurative otitis media, recurrent, bilateral
H65.197	Other acute nonsuppurative otitis media, recurrent, unspecified ear
H65.199	Acute otitis media with effusion
H65.90	Unspecified ear, otitis media with effusion
H65.91	Right otitis media with effusion
H65.92	Left otitis media with effusion
H65.93	Bilateral otitis media with effusion
H66.001	Acute suppurative otitis media without spontaneous rupture of ear drum, right ear
H66.002	Acute suppurative otitis media without spontaneous rupture of ear drum, left ear
H66.003	Acute suppurative otitis media without spontaneous rupture of ear drum, bilateral
H66.004	Acute suppurative otitis media without spontaneous rupture of ear drum, recurrent, right ear

H66.005	Acute suppurative otitis media without spontaneous rupture of ear drum, recurrent, left ear
H66.006	Acute suppurative otitis media without spontaneous rupture of ear drum, recurrent, bilateral
H66.007	Acute suppurative otitis media without spontaneous rupture of ear drum, recurrent, unspecified ear
H66.009	Acute suppurative otitis media without spontaneous rupture of ear drum, unspecified ear
H66.011	Acute suppurative otitis media with spontaneous rupture of ear drum, right ear
H66.012	Acute suppurative otitis media with spontaneous rupture of ear drum, left ear
H66.013	Acute suppurative otitis media with spontaneous rupture of ear drum, bilateral
H66.014	Acute suppurative otitis media with spontaneous rupture of ear drum, recurrent, right ear
H66.015	Acute suppurative otitis media with spontaneous rupture of ear drum, recurrent, left ear
H66.016	Acute suppurative otitis media with spontaneous rupture of ear drum, recurrent, bilateral
H66.017	Acute suppurative otitis media with spontaneous rupture of ear drum, recurrent, unspecified ear
H66.019	Acute suppurative otitis media with spontaneous rupture of ear drum, unspecified ear
H66.40	Suppurative otitis media of unspecified ear, unspecified
H66.41	Suppurative otitis media of right ear, unspecified
H66.42	Suppurative otitis media of left ear, unspecified
H66.43	Suppurative otitis media of both ears, unspecified chronicity
H66.90	Otitis media, unspecified, unspecified ear
H66.91	Otitis media, unspecified, right ear
H66.92	Otitis media, unspecified, left ear
H66.93	Otitis media, unspecified, bilateral
